# SIRT1 is involved in adrenocortical cancer growth and motility

**DOI:** 10.1111/jcmm.16317

**Published:** 2021-03-02

**Authors:** Adele Chimento, Arianna De Luca, Marta Claudia Nocito, Sara Sculco, Paola Avena, Davide La Padula, Lucia Zavaglia, Rosa Sirianni, Ivan Casaburi, Vincenzo Pezzi

**Affiliations:** ^1^ Department of Pharmacy and Health and Nutritional Sciences University of Calabria Arcavacata di Rende, Cosenza Italy

**Keywords:** adrenocortical cancer, ERα, IGF1R, sirt1, sirtinol

## Abstract

Adrenocortical cancer (ACC) is a rare tumour with unfavourable prognosis, lacking an effective treatment. This tumour is characterized by IGF‐II (insulin‐like growth factor II) overproduction, aromatase and ERα (oestrogen receptor alpha) up‐regulation. Previous reports suggest that ERα expression can be regulated by sirt1 (sirtuin 1), a nicotinamide adenine dinucleotide (NAD+)‐dependent class III histone deacetylases that modulates activity of several substrates involved in cellular stress, metabolism, proliferation, senescence, protein degradation and apoptosis. Nevertheless, sirt1 can act as a tumour suppressor or oncogenic protein. In this study, we found that in H295R and SW13 cell lines, sirt1 expression is inhibited by sirtinol, a potent inhibitor of sirt1 activity. In addition, sirtinol is able to decrease ACC cell proliferation, colony and spheroids formation and to activate the intrinsic apoptotic mechanism. Particularly, we observed that sirtinol interferes with E2/ERα and IGF1R (insulin growth factor 1 receptor) pathways by decreasing receptors expression. Sirt1 involvement was confirmed by using a specific sirt1 siRNA. More importantly, we observed that sirtinol can synergize with mitotane, a selective adrenolitic drug, in inhibiting adrenocortical cancer cell growth. Collectively, our data reveal an oncogenic role for sirt1 in ACC and its targeting could implement treatment options for this type of cancer.

## 1 INTRODUCTION

ACC is a rare tumour of the adrenal gland, extremely invasive and with relatively short life expectancies.^1^ This tumour is characterized by considerable morphological, clinical and genetic heterogeneity limiting the availability of specific pharmacological treatments.^2^ Molecular studies identified a correlation between ACC onset and genetic mutations, particularly involving TP53, CTNNB1 (β‐catenin) and IGF‐II genes.^3^ Recently, genomic characterizations of ACC expanded the list of known mutations including also those on PRKAR1A (protein kinase cAMP‐dependent regulatory type I alpha), RPL22 (ribosomal protein L22), TERF2 (telomere specific protein 2), CCNE1 (cyclin E1) and NF1 (neurofibromatosis type 1) genes ^4^ and revealed high heterogeneity and histotype‐specific genomic profiles.^5^ The IGF‐II overexpression is the most widespread molecular event that occurs in 90% of the ACC patients and causes an autocrine mitogenic effect through activation of different signalling pathways mediated by IGF1R.^6^ In addition, this tumour has an increased aromatase and ERα expression^7^ and a cross‐talk between ERα and IGF1R pathways has been demonstrated.^8^ In particular, in H295R cells, IGF‐II‐IGF1R signalling activation leads to an increased aromatase expression and, as a consequence, local oestrogens production, which in turn, through an autocrine mechanism, activates ERα and downstream molecular events that overlap with IGF1R signal.^8^ Moreover, the use of hydroxytamoxifen, an active metabolite of the oestrogen antagonist tamoxifen, decreases IGF1R expression and counteract E2‐ and IGF‐II‐induced ACC cell growth, both in vitro and in vivo.^8^ These results revealed the central role of the oestrogenic pathway and supported the possibility of using anti‐oestrogens as treatment for ACC.

Currently, the main therapeutic approach against ACC is represented by surgery followed by adjuvant drug treatment with mitotane administered as monotherapy or in combination with doxorubicin, vincristine and etoposide in order to decrease recurrence risk.^2^ However, these therapeutic approaches are ineffective for all forms of ACCs. Several clinical trials evaluated effectiveness of targeted therapy, however with discouraging results.^9^, ^10^ For this reason, the widening on knowledge regarding the molecular pathways involved in ACC progression represents a necessary step to develop new therapeutic strategies against this tumour.

It has been reported that ERα expression can be regulated by sirt1,^11^ a mammalian NAD+‐dependent deacetylase that belongs to HDACs (class III histone deacetylases).^12^ Sirt1 targets specific histones such as histone H1 at lysine 26 (H1K26), H3K9 and H4K16, regulating chromatin silencing and heterochromatin formation, and several non‐histone proteins including p53, FOXOs (class O forkhead box transcription factors), PPAR‐γ (peroxisome proliferator‐activated receptor‐gamma), CREB (cyclic AMP‐responsive element‐binding protein), FXR (farnesoid X receptor), HIF‐1α (hypoxia‐inducible factor‐1α), Myc, 5'‐deoxyribose‐5‐phosphate lyase Ku70, E2F1, NF‐kB (nuclear factor kappa‐light‐chain‐enhancer of activated B cells), PGC‐1α (peroxisome proliferator‐activated receptor‐g coactivator‐1α), LXR (liver X receptor) and other, modulating their activity, subcellular localization or association with other proteins.^12^

Although sirt1 is typically localized in the nucleus, a nucleocytoplasmic shuttling has been reported clarifying its different distribution in various tissues and cells affecting its function.^13^ In mammalian cells, sirt1 plays an important role in various biological process such as gene transcription, energy and lipid metabolism, insulin secretion and ageing.^14^ Moreover, sirt1 is involved in neurodegenerative, immune/autoimmune, age‐ and heart‐related disease ^14^ and in cancer.^15^ Particularly, sirt1 has a role in cell proliferation, migration, invasion, genome stability, senescence and apoptosis exerting pro‐ and anti‐tumour activity.^16^ The involvement of sirt1 in genome stability, through chromatin regulation and DNA repair, explains its role as tumour suppressor.^17^

In contrast, other reports indicates that sirt1 promotes cell proliferation and metastasis in a variety of cancers including pancreatic,^18^ hepatocellular,^19^ prostate,^20^ lung,^21^ breast,^22^ cervical,^23^ endometrial^24^ and ovarian^25^ carcinoma.

In this effort, sirtinol (2‐[(2‐Hydroxynaphthalen‐1‐ylmethylene)amino]‐N‐(1‐phenethyl) Benzamide), a potent inhibitor for sirt1^26^ exhibited anti‐proliferative effects in several human cancer cells^27^ and was proposed as anti‐tumour agent.^28^

Starting from these observations, aim of this study was to evaluate the role of sirt1 in ACC. To this purpose, we targeted sirt1 pharmacologically and by RNA‐silencing to evaluate the effects on adrenocortical cancer cell proliferation and metastatic potential.

## 2 MATERIALS AND METHODS

### 2.1 Cell cultures

Adrenocortical tumour cells (H295R and SW13 cells) were purchased from the American Type Culture Collection (ATCC, Rockville, MD). H295R cells were maintained as previously described.^29^ SW13 were maintained in high glucose DMEM (Dulbecco's modified Eagle's medium) (Thermo Fisher Scientific, Monza, Italy) supplemented with 10% foetal bovine serum, 1% glutamine and 1% penicillin‐streptomycin (Sigma‐Aldrich Srl., Milan, Italy). All cells were maintained at 37˚C in a humidified atmosphere of 95% air and 5% CO2. Cell monolayers were subcultured into 6‐well plate for protein and RNA extraction (1 x 10^6^ cells/plate), into 12‐multi‐well for colony formation (2 x 10^3^ cells/well) and wound healing assay (3 x 10^5^ cells/well) and into 48‐multi‐well for MTT assay (2 x 10^4^ cells/well). Doubling time for SW13 cells is about 24 hours and for H295R cells is 48‐72‐hours: this difference has been considered in cell experiments.^30^ H295R and SW13 cells were maintained in complete medium for 48 and 24 hours, respectively, and then treated with sirtinol (Sigma‐Aldrich) in complete medium.

### 2.2 Reagents and antibodies

DMEM‐F12 (Dulbecco's modified Eagle's medium‐F12) was purchased from Sigma (Sigma‐Aldrich). FBS (foetal bovine serum), ITS (insulin‐transferrin‐selenium), L‐glutamine, DMSO (dimethyl sulfoxide), MTT (3‐[4,5‐dimethylthiazol‐2‐yl]‐2,5‐diphenyltetrazoliumbromide), penicillin, streptomycin and sirtinol were purchased from Sigma (Sigma‐Aldrich). Antibodies against sirt1, ERα, IGF1R, CCND1 (cyclin D1), vimentin, N‐cadherin, bax (bcl2 associated x), bcl‐2 (B‐cell lymphoma 2), parp‐1 (poly(ADP‐ribose) polymerase 1), cytochrome c, pCREB‐1^Ser133^ (phospho cyclic AMP response element binding) and GAPDH (glyceraldehyde 3‐phosphate dehydrogenase) were purchased from Santa Cruz (Santa Cruz Biotechnology, Inc, Heidelberg, Germany). Antibody against VDAC1 (voltage‐dependent anion‐selective channel protein 1)/porin) was purchased from Abcam (Abcam, Cambridge, UK). HRP (Horseradish peroxidase)‐conjugated secondary antibodies were purchased from Bethyl (Bethyl Laboratories, Montgomery, TX, USA) and ECL (Electrochemiluminescence) Western blotting detection system from Santa Cruz (Santa Cruz Biotechnology, Santa Cruz CA, USA). The TRIzol reagent, High Capacity cDNA Reverse Transcription Kit and PowerUp™ SYBR™ Green Master Mix were purchased from Thermo Fisher Scientific (Thermo Fisher Scientific).

### 2.3 Cell viability assay

The effect of sirtinol on cell viability was measured using MTT assay as previously described.^29^, ^31^

### 2.4 RNA silencing

Cells were subcultured in 6‐multi‐well plates (2.5 × 10^5^ cells/well) for Western blot analysis or in 24‐multi‐well plates (0.5 × 10^5^ cells/well) for cell viability assay. The next day, as recommended by manufacturer, cells were transfected with control siRNA (siRNA scrambled) or sirt1 siRNA (Thermo Fisher Scientific) in complete medium using lipofectamine RNAiMAX transfection reagent (Thermo Fisher Scientific) for a total of 72 hours.

### 2.5 Spheroids cultures

A single cell suspension was prepared using 1x Trypsin‐EDTA (ethylenediaminetetraacetic acid) solution (Sigma‐Aldrich) and manual disaggregation (21 gauge needle).^32^ Cell were seeded in non‐adherent conditions as described by De luca and collegues.^33^

### 2.6 Colony Formation Assay

Two thousand cells were seeded in 12‐well plates and allowed to grow out in the absence or presence of different sirtinol concentrations for 14 (H295R) or 7 (SW13) days. Colonies were stained with 0.05% Coomassie Blue in methanol/water/acetic acid (45:45:10, v/v). Colony number was assessed using Image J (NIH) and normalized to untreated cells.

### 2.7 Wound healing assay

Cells were grown in 12‐well plates until about 80‐90% confluency was reached and then a 10‐μL pipette tip was used to create a scratch/wound with clear edges across the width of a well. Wells were treated either with vehicle (DMSO) or 40 μmol/L sirtinol. Photographs were acquired with Olympus CKX53 microscope at 0 hours or 24 hours. All experiments were performed in triplicates.

### 2.8 Transwell migration assay

The transwell inserts (8 μm pore size, 24‐well plate, Corning Costar, Cambridge, MA) were used to evaluate cell migration ability. The cells (5 × 10^4^/well) were seeded in the boyden insert and solvent or sirtinol were added in the well; the cells were allowed to migrate across the membrane for 24 hours. At the end of the experiment, migrated cells were stained with Coomassie Brilliant Blue solution for 10 minutes and counted under an inverted microscope (Olympus CKX53).

### 2.9 RNA extraction, reverse transcription and qPCR

The RNA extraction was performed as previously described.^29^ One (for H295R) or 2 (for SW13) micrograms of total RNA were reverse‐transcribed in a final volume of 50 μL using the High Capacity cDNA Reverse Transcription Kit (Thermo Fisher Scientific); cDNA was diluted 1:2 in DNAse and RNAse free water. Primer sequences are shown in Table 1. PCR reactions were performed in the QuantStudio^Tm^ 3 Real Time PCR System (Thermo Fisher Scientific) using 0.3 (for H295R) or 0.6 (for SW13) μmol/L of each primer. PowerUp™ SYBR™ Green Master Mix (Thermo Fisher Scientific) with the dissociation protocol was used for gene amplification; negative controls contained water instead of first‐strand cDNA. Each sample was normalized to its 18S rRNA (18S) content. Final results were expressed as n‐fold differences relative to a calibrator and calculated using the ΔΔCt method.

**TABLE 1:** Primers oligonucleotide sequences. The indicated primers oligonucleotide sequences were used for the amplification of following genes: SIRT1: sirtuin 1; ESR1: oestrogen receptor alpha; CCND1: cyclin D1; IGF1R: insulin growth factor receptor 1; 18S: 18S ribosomal RNA

**Gene**	**Forward primer**	**Reverse primer**
SIRT1	CAGTGGCTGGAACAGTGAGA	AGCGCCATGGAAAATGTAAC
ESR1	CACCATTGATAAAAACAGGAGGAA	CTCCCTCCTCTTCGGTCTTTTC
CCND1	CACGCGCAGACCTTCCG	ATGGAGGGCGGATTGGAA
IGF1R	AAGGCTGTGACCCTCACCAT	CGATGCTGAAAGAACGTCCAA
18S	CGGCGACGACCCATTCGAAC	GAATCGAACCCTGATTCCCCGTC

### 2.10 Western blot analysis

RIPA lysis buffer was used to lysate cells.^34^ Western blot analysis was performed using equal quantity of protein. Protein concentration was determined by Bradford method (Sigma‐Aldrich) and equal amounts were subjected to Western blot analysis. All membranes were incubated for 12 hours at 4°C with antibodies against sirt1, ERα, IGF1R, CCND1, vimentin, N‐cadherin, bax, bcl‐2, parp1 and cytochrome c (all from Santa Cruz Biotechnology, Santa Cruz CA, USA). Membranes were incubated with HRP‐conjugated secondary antibodies (Bethyl Laboratories, Montgomery, TX, USA), and immunoreactive bands were visualized with the ECL Western blotting detection system (Santa Cruz Biotechnology, Santa Cruz CA, USA). GAPDH (Santa Cruz Biotechnology, Santa Cruz CA, USA) or VDAC1/porin (Abcam, Cambridge, UK) antibodies were used as a loading control.

### 2.11 Cytochrome c detection

Cytochrome c (cyt c) detection in mitochondrial and cytoplasmic fractions was performed as previously reported.^29^

### 2.12 Terminal deoxynucleotidyl transferase dUTP nick‐end labeling (TUNEL) assay

Cells seeded onto glass coverslips were treated with sirtinol for 48 hours, washed with PBS and then fixed in 4% paraformaldehyde for 15 minutes at room temperature. The cells were subsequently washed with PBS and then soaked for 20 minutes in PBS containing 0.25% of Triton X‐100. After two washes with deionized water, slides were incubated with TdT (terminal deoxynucleotidyl transferase) enzyme and EdUTP (5‐ethynyl‐2'‐deoxyuridine 5'‐triphosphate) overnight at room temperature, using Click‐iT TUNEL Alexa Fluor Imaging Assay (Thermo Fisher Scientific). The next day cells were incubated with a solution containing Alexa Fluor azide for 30 minutes at room temperature and then stained with DAPI (4',6‐diamidino‐2‐phenylindole) solution in order to analyse the nuclear morphology. Cells were observed under a fluorescence microscope (Olympus BX41) with a 20X objective.

### 2.13 Phase‐contrast microscopy for morphological evaluation

H295R and SW13 cells were seeded into 48‐well plates at a density of 2 x 10^4^ cells/well, and then transfected with a specific sirt1 siRNA. Additionally, cells were treated with sirtinol (40 μmol/L) and mitotane (10 μmol/L) alone or in combination for 72 hours. Cells were observed under an inverted phase contrast microscope (Olympus CKX53) with a 10X objective.

### 2.14 Statistics

Statistical analyses were performed as previously indicated.^29^ Coefficient of drug interaction (CDI) was calculated as: CDI = AB/(A × B). AB represents the percentage of viable cells remaining after the treatment with the combined drugs, while A and B represent the percentage of viable cells remaining after single treatments. CDI < 1 indicates a synergistic effect, CDI = 1 indicates an additive effect, CDI > 1 indicates antagonism.

## 3 RESULTS

### 3.1 Sirt1 inhibition exerts anti‐proliferative effects in human adrenocortical cancer cells

**FIGURE 1:**
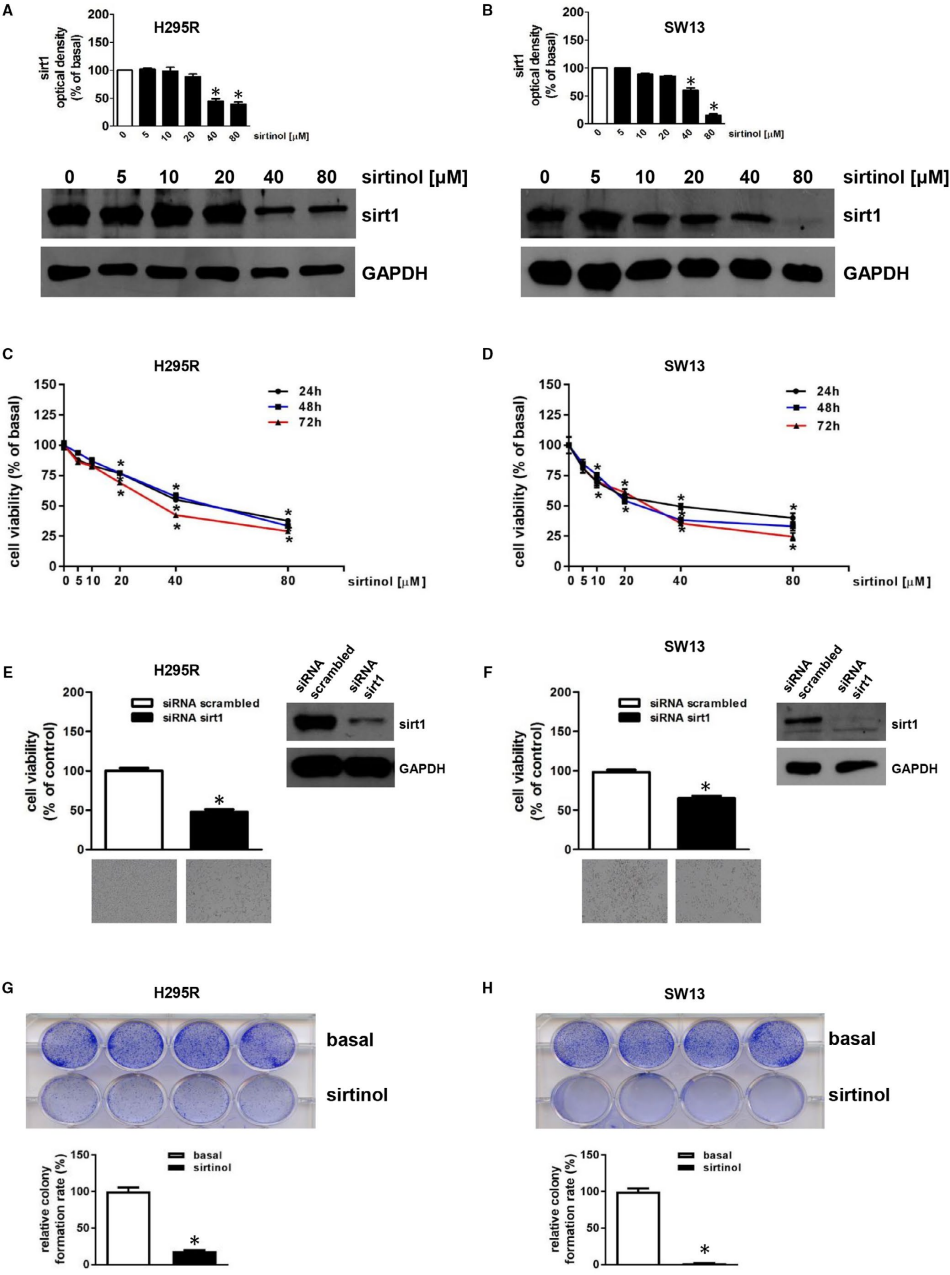
Sirtinol or sirt1 gene silencing reduce H295R and SW13 cell growth. (A,B) H295R (A) or SW13 (B) cells were untreated (0) or treated with increasing doses (5; 10; 20, 40, 80 μmol/L) of sirtinol for 24 h. Total protein extracts were examined by Western blotting analysis for sirt1 expression; GAPDH was used to normalize protein expression. Blots are from one experiment representative of three with similar results. H295R (C) or SW13 (D) cells were untreated (0) or treated with increasing doses (5; 10; 20, 40, 80 μmol/L) of sirtinol for 24, 48 and 72 h. Cell viability was evaluated by MTT assay (*P < .05 vs 0). (E,F) MTT assay was performed on H295R (E) or SW13 (F) cells transfected for 72 h with control siRNA (siRNA scrambled) or with siRNA for sirt1 (siRNA sirt1). Results were expressed as mean ± SE of three separate experiments (*P < .05 vs siRNA scrambled). The insert in E or F illustrates a Western blot of proteins from H295R or SW13, respectively, assessing the expression of sirt1 in the presence of siRNA scrambled or siRNA sirt1. GAPDH was used to normalize protein expression. Below images represent the morphological changes of H295R (E) or SW13 (F) cells after sirt1 silencing. These were observed by an Inverted Optic Microscope (magnification X100) and are from a representative experiment. (G,H) Colony formation assay was performed on H295R (G) or SW13 (H) cells (2000 cells/well) plated in the absence (basal) or presence with sirtinol (40 μmol/L). Relative colony formation rate was evaluated 14 (for H295R) or 6 (for SW13) days later (*P < .05 vs basal). Upper images are from a representative experiment

We first treated cells with increasing concentrations of sirtinol in order to evaluate effects on sirt1. Using Western blot analysis, we revealed that 40 µmol/L sirtinol for 24 hours significantly decreased sirt1 protein expression in H295R (Figure 1A) and SW13 cells (Figure 1B). The same doses were tested on time course experiments to evaluate effects on cell viability, highlighting a clear inhibitory effect on both H295R (Figure 1C) and SW13 (Figure 1D) cells. Sirt1 gene expression breakdown with a specific siRNA (siRNAsirt1) showed a significant reduction in protein content compared to the control (siRNA scrambled) cells (insert, Figure 1E and F) after 72 hours. Sirt1 silencing was able to inhibit H295R (Figure 1E) and SW13 (Figure 1F) cell proliferation by 50% and 40%, respectively. Additionally, sirtinol suppressed the colony‐forming ability of both cell lines (Figure 1G and H).

### 3.2 Sirt1 inhibition induces apoptosis in human adrenocortical cancer cells

**FIGURE 2:**
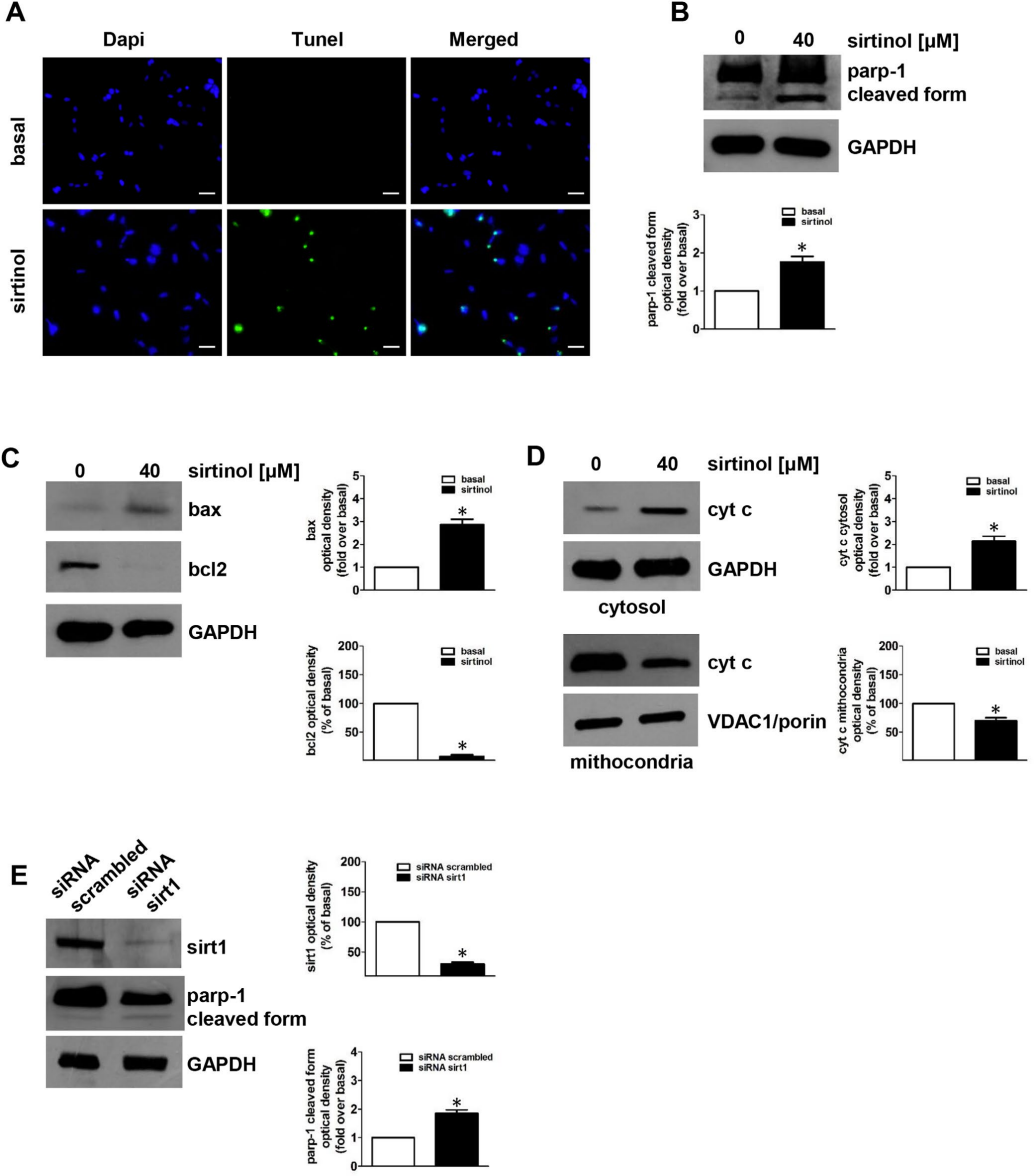
Sirtinol or sirt1 gene silencing induce apoptosis in adrenocortical cancer cells. (A) TUNEL assay was performed on H295R cells, which were previously untreated (basal) or treated with sirtinol (40 μmol/L) for 48 h. Nuclei counterstaining was executed employing DAPI. Fluorescent signal was observed under a fluorescent microscope (magnification X200). Scale bar= 25µm. Images are from a representative experiment. (B‐D) H295R cells were treated with sirtinol (40 μmol/L) for 24 h. Parp‐1(B), bax and bcl‐2 (C) were evaluated by Western blot. (B, below panel; C right panels) Graphs correspond to means of standardized optical densities from three experiments, bars correspond to SE (*P < .05 vs basal). Results are expressed as % of inhibition or fold induction versus basal. Western blot analyses of cytochrome c (cyt c) (D) were performed on cytosolic and mitochondrial protein fractions. Blots are from one experiment representative of three with similar results. GAPDH and VDAC1/porin were used as a loading control for cytosolic and mitochondrial proteins, respectively. (D, right panels) Graphs correspond to means of standardized optical densities from three experiments, bars correspond to SE (*P < .05 vs basal). Results are expressed as % inhibition or fold induction respect to basal. (E) H295R cells were transfected for 72 h with control siRNA (siRNA scrambled) or with siRNA for sirt1 (siRNA sirt1). Parp‐1 and sirt1 were evaluated by Western blot. Blots are from one experiment representative of three with similar results. GAPDH was used to normalize protein expression. (E, right panels) Graphs correspond to means of standardized optical densities from three experiments, bars correspond to SE (*P < .05 vs basal). Results are expressed as % inhibition or fold induction respect to basal

In order to verify if the reduction in cell viability after sirt1 depletion was associated with an apoptotic mechanism, cells were exposed to sirtinol for 48 hours and subjected to TUNEL assay. Sirtinol induced apoptosis in H295R cells as showed by an increased number of TUNEL‐positive cells (green) (Figure 2A). To specify the apoptotic mechanism activated by sirtinol, we measured the expression of different apoptototic‐related proteins by Western blot analysis. Sirtinol significantly increased parp‐1 cleavage (Figure 2B) and bax expression while decreased bcl‐2 content (Figure 2C). It was suggested that cytosolic translocation of cytochrome c is a crucial event in the mitochondria‐dependent apoptotic pathway. Consequently, we investigated if cytochrome c was released into the cytosol after sirtinol treatment. Cytosolic and mitochondrial protein extraction was performed and analysed by Western blot assay (Figure 2D). Cells treated with sirtinol showed increased cytochrome c levels in the cytosolic compartment while decreased in the mitochondrial fraction (Figure 2D). Parp‐1 activation was also observed in the presence of sirt1 siRNA (Figure 2E). Apoptosis was activated by sirtinol also in SW13 cells, as evidenced by parp‐1 cleavage (Figure S1A), increase in bax and decrease in bcl‐2 protein expression (Figure S1B) and mitochondrial cytochrome c release (Figure S1C). In addition, sirt1 silencing led to parp‐1 cleavage (Figure S1D).

### 3.3 Sirt1 inhibition reduces motility of human adrenocortical cancer cells

**FIGURE 3:**
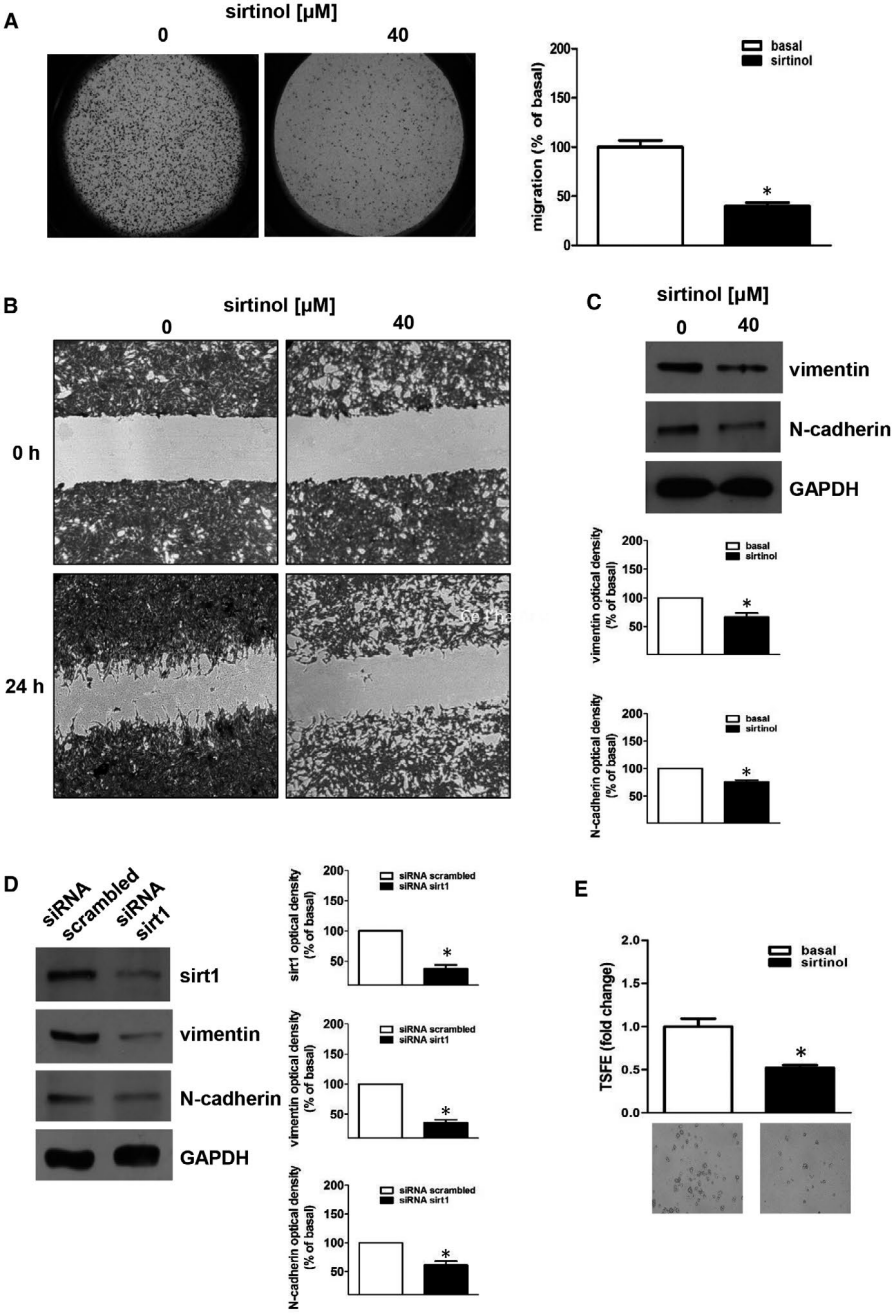
Sirtinol or sirt1 gene silencing inhibit H295R migration and invasion. (A, B) H295R cells were untreated (basal, 0) or treated with sirtinol (40 μmol/L) for 24 h. Transwell migration (A) and wound healing (B) assays were performed as described in ‘Materials and Methods’. Images are from a representative experiment (A, magnification X20; B, magnification X100). (A) In the transwell migration assay, sirtinol was added in the lower compartment. After 24 h of incubation, migrated cells to the lower surfaces of the membranes were observed under a microscope and then counted. Right graph represents the mean ± SE of three independent experiments of migrated cells number expressed setting untreated cells as 100% (basal) (*P ≤ .05 vs basal). (C,D) Western blot analyses of vimentin, N‐cadherin and sirt1 were executed on H295R cells, which were previously untreated (0) or treated with sirtinol (40 μmol/L) for 24 h (C) or transfected for 72 h with control siRNA (siRNA scrambled) or with siRNA for sirt1 (siRNA sirt1) (D). Blots are from one experiment representative of three with similar results. GAPDH was used to normalize protein expression. (C, below panels; D, right panels) Graphs correspond to means of standardized optical densities from three experiments, bars correspond to SE (*P < .05 vs basal). Results are expressed as % inhibition respect to basal. (E) H295R cells were plated on low‐attachment plates and then left untreated (basal) or treated with sirtinol (40 μmol/L). Tumour spheres formation efficiency (TSFE) was evaluated 5 d later (*P < .05 vs basal). Images below graphs are from a representative experiment (magnification X200)

To examine the effects of sirtinol on migratory and invasive properties of H295R cells, transwell migration and wound healing assays were performed. Sirtinol inhibits H295R migratory ability (Figure 3A, 3B). To explain the inhibitory effect on cell migration, we evaluated the expression levels of key genes involved in the acquisition of mesenchymal phenotype. N‐cadherin and vimentin were reduced by sirtinol (Figure 3C) and siRNA for sirt1 (Figure 3D). These results suggest that sirt1 has a role in regulating expression of genes involved in ACC cell motility

We also evaluated adrenocortical cancer cells ability to grow in anchorage‐independent manner forming 3‐dimensional spheres. This model system enriches spheres of cancer stem cells and progenitor cells and more closely mimics tumours in vivo.^33^ When H295R cells were grown as spheroids in the presence of sirtinol, we observed a substantial decrease in spheres number (Figure 3E). Altogether, these data indicate that sirt1 functions as metastatic promoter in ACC.

### 3.4 Sirt1 is part of the genomic and non‐genomic ERα actions

**FIGURE 4:**
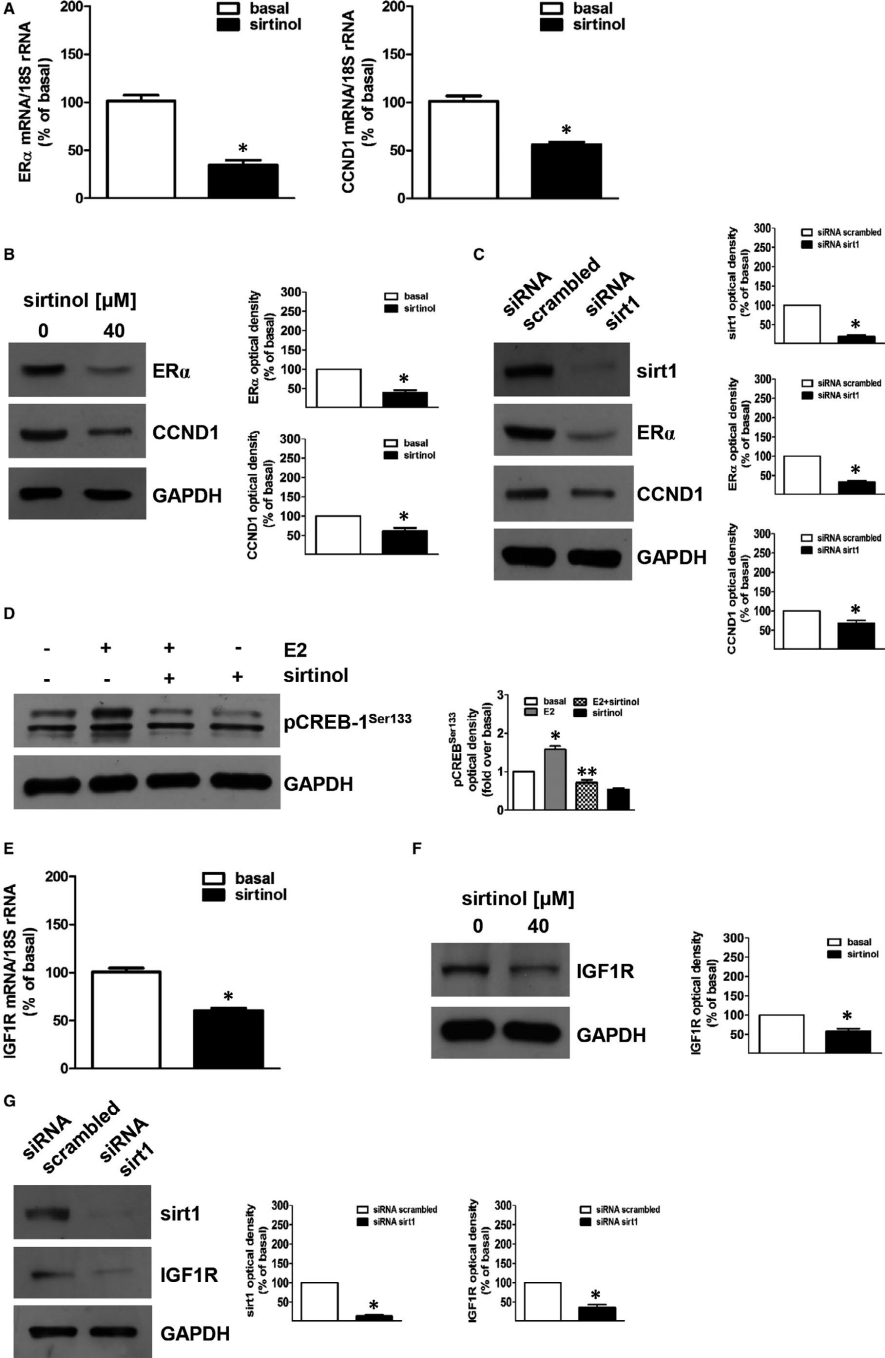
Sirtinol or sirt1 gene silencing inhibit ERα expression and oestrogen‐dependent pathways. (A,E) H295R cells were untreated (basal, 0) or treated with sirtinol (40 μmol/L) for 24 h. The mRNA was extracted and analysed by real‐time RT‐PCR. Gene expression of ERα and cyclin D1 (CCND1) (A) or IGF1R (E) in every sample was standardized to 18S rRNA content. N‐fold differences of gene expression compared to calibrator (untreated sample) were used to graph final results. Data correspond to mean ± SE of values from at least three separate RNA samples (*P < .05 vs calibrator). (B,F) H295R cells were untreated (basal, 0) or treated with sirtinol (40 μmol/L) for 24 h. Total protein extracts were analysed by Western blot analysis for the expression of ERα and CCND1 (B) or IGF1R (F); GAPDH was used to normalize protein expression. Blots are from one experiment representative of three with similar results. (B,F right panels) Graphs correspond to means of standardized optical densities from three experiments, bars correspond to SE (*P < .05 vs basal). Results are expressed as % inhibition respect to basal. (C,G) H295R cells were transfected for 72 h in the presence of control siRNA (siRNA scrambled) or siRNA for sirt1 (siRNA sirt1). Total protein extracts were analysed by Western blot analysis for the expression of sirt1, ERα and CCND1 (C) or sirt1 and IGF1R (G); GAPDH was used to normalize protein expression. Blots are from one experiment representative of three with similar results. (C,G right panels) Graphs correspond to means of standardized optical densities from three experiments, bars correspond to SE (*P < .05 vs basal). Results are expressed as % of inhibition versus basal. (D) H295R cells were untreated (−) or treated with sirtinol (40 μmol/L) for 24 h. Then, cells were treated with estradiol (E2) (100 nmol/L) for 1 h as indicated. Total protein extracts were analysed by Western blot analysis for the expression of pCREB‐1^Ser133^; GAPDH was used to normalize protein expression. Blots are from one experiment representative of three with similar results. (D, right panel). Graphs correspond to means of standardized optical densities from three experiments, bars correspond to SE (*P < .05 vs basal; **P < .05 vs E2). Folds induction versus basal or E2 were used to express results

In order to clarify how sirt1 regulates ACC growth, we investigated its role in E2/ERα and IGF1R signalling. Using a quantitative real‐time PCR analysis, we observed that inhibition of sirt1 reduces mRNA levels of ERα and CCND1, a major ERα target gene involved in cell cycle regulation,^35^ in H295R (Figure 4A) as well as in SW13 cells (Figure S2A). Similar effects were reproduced at the protein level by both sirtinol and sirt1 siRNA in H295R (Figure 4B,C) and SW13 (Figure S2B,C) cells, confirming a role for sirt1 in ERα genomic actions in ACC. Our previous data demonstrated that ERα, in a non‐genomic fashion, is involved in CREB phosphorylation.^8^ Our results showed that E2 caused a strong activation of CREB, confirming previous report ^8^; this effect was abrogated by co‐treatment with sirtinol in both H295R (Figure 4D) and SW13 cells (Figure S2D).

CREB is a transcription factor involved in IGF1R expression.^8^ Our results indicated that sirtinol treatment decreases IGF1R mRNA and protein expression levels in both H295R (Figure 4E,F) and SW13 (Figure S2E,F) cells. Similar effects were observed by sirt1 gene silencing in H295R (Figure 4G) and SW13 cells (Figure S2G). These data suggest that sirt1 modulates E2/ERα and IGF1R pathways.

### 3.5 Sirtinol potentiates mitotane effects in human adrenocortical cancer cell growth

**FIGURE 5:**
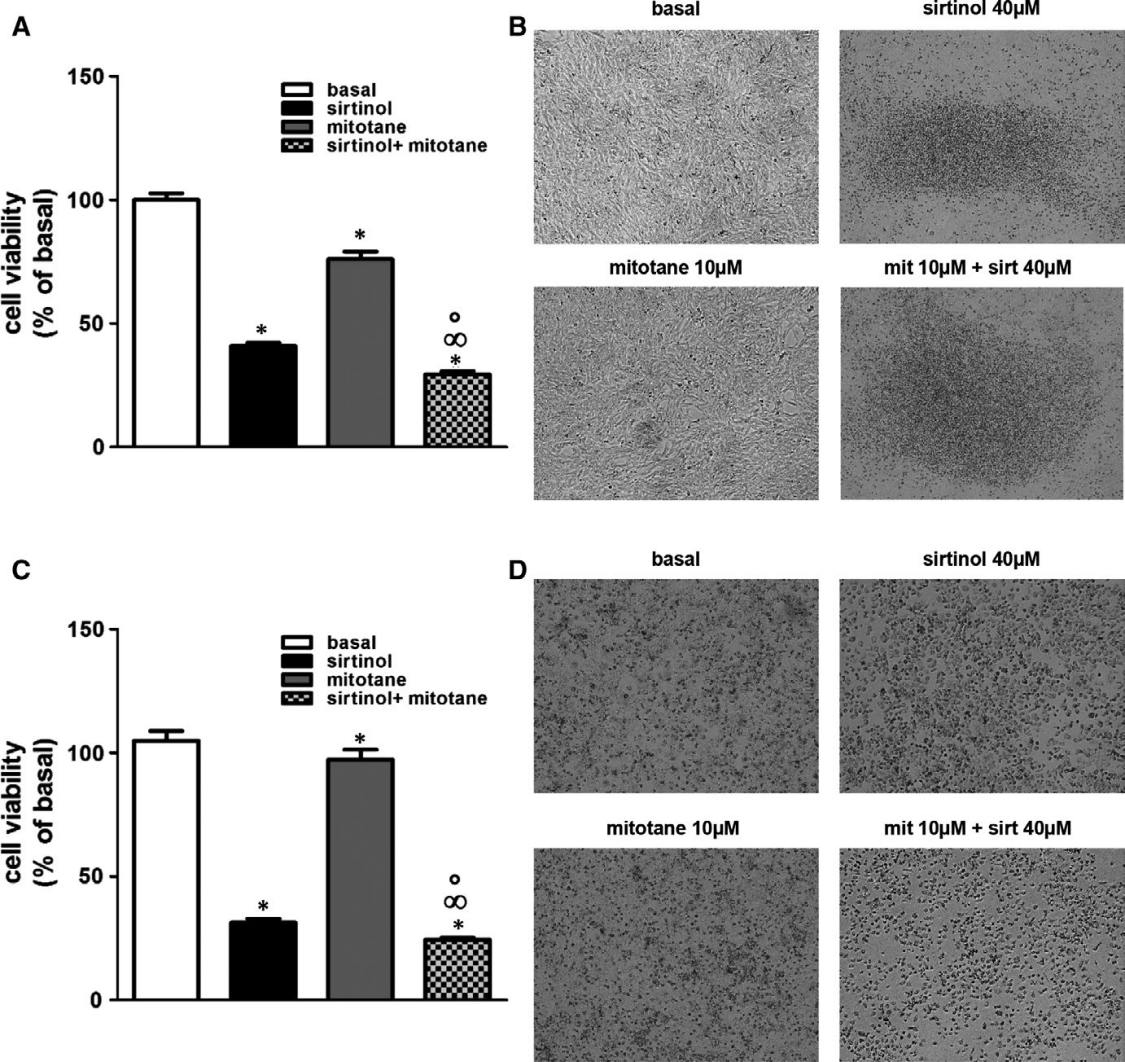
Sirtinol and mitotane display synergistic inhibitory effects on adrenocortical cancer cell growth. H295R (A) and SW13 (C) cells were left untreated (basal) or treated with sirtinol (40 μmol/L) and mitotane alone (10 μmol/L) or in combination for 72 h. Cell viability was evaluated by MTT assay (*P < .05 vs basal; °*P* < .05 vs sirtinol; ∞*P* < .05 vs mitotane). (B, D) Morphological changes of H295R (B) and SW13 (D) cells after treatments. Cell images were acquired using an Inverted Microscope (magnification X100)

We finally conducted experiments to establish if sirtinol is able to potentiate the effects of mitotane on cell growth. As evidenced in Figure 5, 10 µmol/L mitotane was able to reduce H295R cell viability by 20%, when combined with sirtinol 40 µmol/L inhibition increased to about 60% (Figure 5A). The coefficient of drug interaction (CDI) method^36^ was then used to evaluate the effects of sirtinol and mitotane on H295R cell viability. Based on CDI value (0.95), at the tested doses, the two drugs exert a synergistic effect. Figure 5B shows H295R cell morphology after 72‐hours exposure to sirtinol, mitotane and combination of the two drugs. Treatments decrease attachment to the plate, with the combination producing a more pronounced effect. It can be appreciated the presence of spherical dead cells, more abundant with sirtinol plus mitotane, confirming data derived from CDI (Figure 5B). Effects of combined mitotane and sirtinol treatment were also studied in SW13 cells. As showed in Figure 5C, sirtinol combined with the subtherapeutic 10 µmol/L dose of mitotane was able to potentiate its effects (CDI value: 0.80) (Figure 5C). Moreover, sirtinol 40 µmol/L has pronounced effects on cells viability compared to mitotane, but the combination of the two drugs has more profound effects on cell death and detachment from the plate (Figure 5D). Collectively these data, support the hypothesis of synergistic anti‐proliferative effects of mitotane and sirtinol.

## 4 DISCUSSION

In this study, for the first time, we evidenced a role for sirt1 in ACC cell growth and EMT (epithelial/mesenchymal transition). Sirt1 functions in tumour have been widely discussed, indicating an effect as both a tumour suppressor and oncogenic factor, depending on the cell context. Literature data converged in asserting that sirt1 may modulate a delicate balance between suppression and promotion of tumorigenesis, depending on its level of activity, spatial and temporal distribution and the stage of tumorigenesis.^37^

In this study, we evaluated sirt1 expression and function in H295R and SW13 adrenocortical cancer cells. We demonstrated that pharmacological inhibition of sirt1 by sirtinol reduced H295R and SW13 cell viability in a time‐ and dose‐dependent manner and decreased cell ability to form colonies. Similarly, gene silencing with a specific siRNA produces the same inhibitory effects on cell viability, confirming the oncogenic function of this protein. In support to its oncogenic role, it has been reported that sirt1 deacetylates and inactivates tumour suppressors promoting cell proliferation and angiogenesis and blocking apoptosis.^27^, ^38^ Data from a study in human breast cancer, MCF‐7, and lung cancer, H1299, cells show that sirtinol induced senescence‐like growth arrest characterized by induction of β‐galactosidase activity and increased expression of plasminogen activator inhibitor.^27^ In human colorectal carcinoma cells, sirt1 deficiency attenuated viability in vitro and tumorigenicity in vivo.^39^ Additionally, another paper reported how in human epithelial cancer cells derived from colorectal, breast and cervical carcinomas, sirt1 silencing induces growth arrest and/or apoptosis.^40^ Similarly, in cutaneous T‐cell lymphoma sirt1 knockdown resulted in reduced cellular metabolism and proliferation, increased apoptosis and PARP‐1 inactivation.^41^

To explore the mechanism responsible for sirtinol anti‐cancer effects in adrenocortical cancer cells, we first confirmed its ability to activate apoptosis. Here, we demonstrated that sirt1 inhibition using sirtinol activates apoptosis, demonstrated by TUNEL assay, increased cytochrome c release into the cytoplasm, up‐regulation of bax, down‐regulation of bcl‐2 and inactivation of parp‐1. Sirtinol ability to produce such effects is in agreement with other previous reports in breast cancer cells. Sirt1 inhibition allows the increase of bax expression, cytochrome c release, decrease of bcl‐2 and activation of apoptosis.^42^, ^43^ In this paper, we also confirmed a role for sirt1 in the EMT, a process in which the loss of non‐mobile epithelial phenotype allows cells to dissolve their cellular junctions and transform into individual and mobile mesenchymal cells leading tumour metastasis.^44^ We demonstrated that the lack of sirt1 interferes with H295R cell motility and migration reducing the expression of some EMT markers such as N‐cadherin and vimentin. These results are in agreement with other reports demonstrating sirt1 involvement in EMT. In triple negative breast cancer, sirt1 induces tumour invasion by targeting EMT‐related pathway.^45^, ^46^ Similarly, sirt1 was found to promote EMT in hepatocellular,^47^ prostate^48^ and oral squamous.^49^ Although sirt1 inhibition modulates expression of EMT protein markers in H295R cells, this event was not observed in SW13 cells. Indeed, SW13 cells can exist in two subtypes, one expressesing vimentin (SW13+) and another lacking expression of this protein (SW13‐)^50^ and for our study we used this subtype. Of note, SW13 cells are a depot in the adrenal of a primary lung cancer, while H295R cells derive from a female affected by a primary adrenocortical carcinoma^30^ and this observation can explain the different characteristics in terms of motility between the two cell lines. It can therefore be postulated that invasion and migration of SW13 relies on other mesenchymal markers such as βIII‐tubulin that is expressed only in SW13 vimentin‐deficient cells.^51^ In fact, βIII‐tubulin confers brain metastatic potential to breast cancer cells by regulating invasion^52^ and Integrin‐Src signalling.^53^ β‐tubulin depletion reduces metastasis via down‐regulation of signalling molecules such as β3 Integrin, p‐FAK and p‐Src in MDA‐MB231 cells.^53^ Furthermore, pathological EMT also shows great complexity depending on the tissue context. In fact, the expression and function of different EMT inducers vary considerably between different types of cancer and therefore can function in a tumour‐type‐specific manner.^54^ Induction of EMT characteristics can result in the expression of stem cell markers and increased ability to form spheres.^55^, ^56^ Starting from our previous results indicating that H295R cells grown in low‐attachment plates undergo anchorage‐independent growth, promoting the growth of 3‐dimensional spheres with the properties of cancer stem cells and progenitor cells,^33^ we demonstrated that sirt1 is able to modulate this mechanism. In fact, sirtinol reduced H295R spheres formation.

In the second part of our study, we wanted to investigate the specific molecular pathway responsible for sirtinol effects. Particularly, evaluating effects on key regulators of ACC, such as ERα and IGF1R in our experimental models. Previous studies demonstrated that aromatase and ERα are overexpressed in ACC,^7^ local oestrogens bind and activate ERα, which through genomic and non‐genomic mechanisms, regulates cell growth.^8^ Here, we demonstrated that loss of sirt1 down‐regulated ERα and its target gene CCND1.^35^ ^,^ ^57^ We found that sirtinol attenuated E2‐dependent CCND1 expression (data not shown). Additionally, sirtinol prevented E2‐dependent CREB activation, highlighting its ability to interfere with ERα non‐genomic action.^8^

Importanly, we demonstrated a synergistic inhibitory action of sirtinol and mitotane on H295R and SW13 cell viability. Mitotane has been clinically used for decades as adjuvant therapy for adrenocortical carcinoma, despite its side effects. Patients achieving plasma mitotane levels above 14 mg/L show a good response rate and an improved survival, however, this concentration cannot be reached in all patients, because of the neurological and gastrointestinal adverse effects.^58^ We evidenced that when used in combination with sirtinol, mitotane dose can be lowered to one‐fifth of the therapeutic dose. In general, treatment strategies combining mitotane with other drugs could increase the response rate of patients, as compared with monotherapy. This can occur because the combination allows synergistic effects that potentially increase the in vivo anti‐neoplastic action, or because the two drugs elicit different mechanisms that increase patients chances to get a clinical benefit from at least one of the two treatments. It was demonstrated that a combination of mitotane and chemotherapeutic drugs might be more effective in ACC treatment.^58^ A recent study indicated that the use of nilotinib, a tyrosine kinase inhibitor, in combination with mitotane inhibited cell viability more significantly then mitotane alone.^59^ In another work, addition of mTOR (mammalian target of rapamycin) inhibitor, sirolimus, to low concentrations of mitotane improved the anti‐proliferative effects exerted by mitotane alone.^60^

Overall, our study proves that targeting sirt1 is sufficient to reduce activity of two major players in ACC: oestrogens and IGF‐II. Additionally, sirtinol ability to synergize with mitotane provides a rationale for further investigating sirtinol effects in vivo on both tumour growth and metastases and opens new perspectives for a different therapeutic approach to targeting this tumour.

## 5 CONCLUSIONS

**FIGURE 6:**
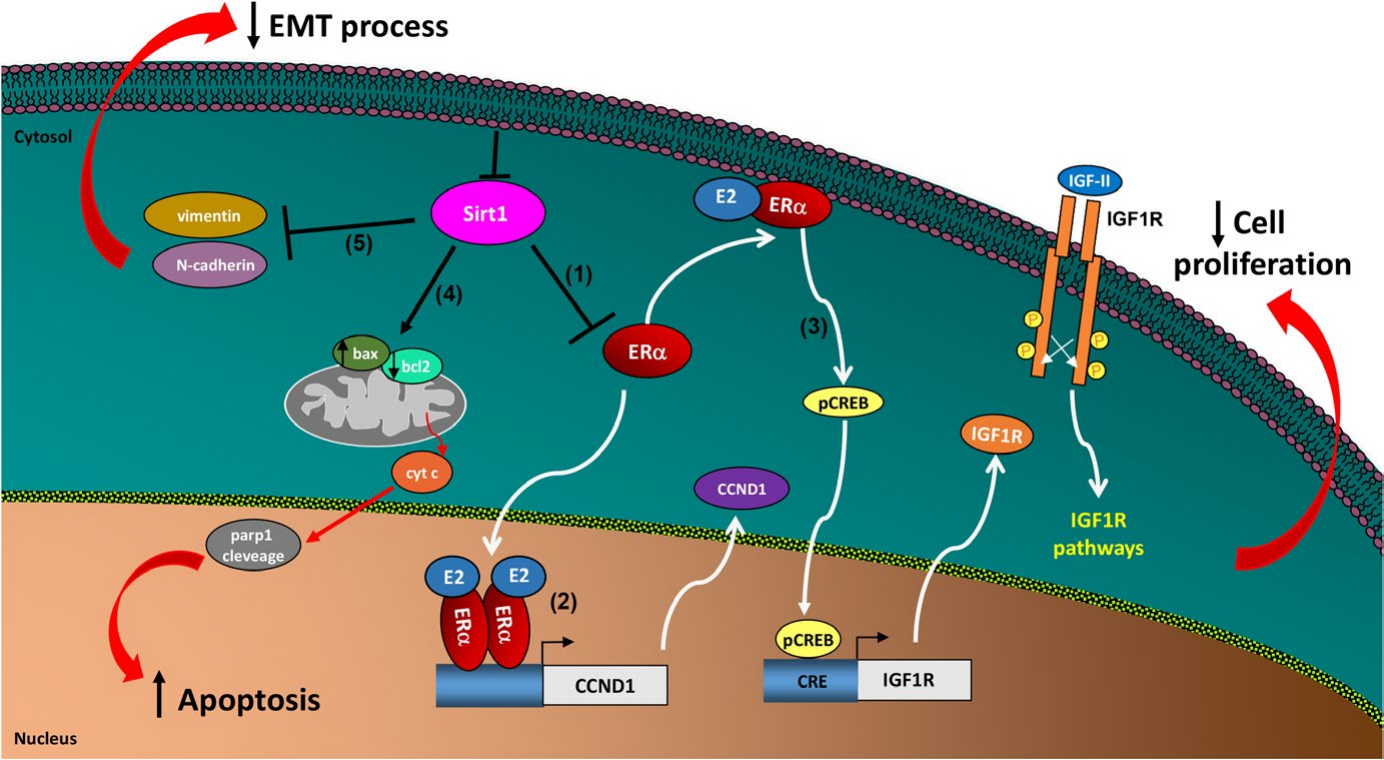
Schematic representation of molecular mechanisms induced by sirt1 inhibition in ACC cells. Sirt1 inhibition reduces ERα expression (1). Reduction of ERα genomic action decreases CCND1 expression (2) influencing ACC cell proliferation. Reduction of ERα non‐genomic action decreases CREB phosphorylation and consequently IGF1R expression (3) influencing ACC cell proliferation. In addition, sirt1 inhibition causes ACC cell death by activating the intrinsic apoptotic pathway (4). Moreover sirt1 depletion reduces ACC cell motility regulating EMT process (5)

In conclusion, in this study we provide evidences regarding the role of sirt1 as an oncogenic and anti‐apoptotic factor in ACC. In particular, we revealed that both sirt1 pharmacological inhibition (sirtinol) and gene silencing reduce proliferation of H295R and SW13 adrenocortical cancer cells by interfering with E2/ERα and IGF1R pathways through the inhibition of several proteins, such as ERα, CCND1 and IGF1R (Figure 6) and activating apoptosis. In addition, we confirmed a role for sirt1 in adrenocortical cancer cell motility and EMT process. The observation that a single drug such as sirtinol is able to block several targets involved in ACC growth and metastasis, together with the discovery that sirtinol can synergize with mitotane in inhibiting tumour growth, opens new perspectives for a different therapeutic approach of ACC. Finally, our results, confirming the oncogenic role of sirt1 in adrenocortical cancer cells, propose it as a useful molecular target against ACC.

## CONFLICT OF INTEREST

The authors declare that they have no conflict of interest.

## AUTHOR CONTRIBUTIONS

**Adele Chimento:** Conceptualization (equal); Data curation (equal); Investigation (equal); Methodology (equal); Validation (equal); Writing‐original draft (equal). **Arianna De Luca:** Investigation (equal); Methodology (equal); Validation (equal). **Marta Claudia Nocito:** Investigation (equal); Methodology (equal); Validation (equal). **Sara Sculco:** Investigation (equal); Methodology (equal); Validation (equal). **Paola Avena:** Investigation (equal); Methodology (equal); Validation (equal). **Davide La Padula:** Investigation (equal); Methodology (equal); Validation (equal). **Lucia Zavaglia:** Investigation (equal); Methodology (equal); Validation (equal). **Rosa Sirianni:** Data curation (equal); Investigation (equal); Methodology (equal); Validation (equal). **Ivan Casaburi:** Data curation (equal); Investigation (equal); Methodology (equal); Supervision (equal); Validation (equal). **Vincenzo Pezzi:** Conceptualization (equal); Data curation (equal); Investigation (equal); Methodology (equal); Supervision (equal); Validation (equal); Writing‐original draft (equal).

## ETHICAL APPROVAL

This article does not contain any studies with human participants or animals performed by any of the authors.

## Supporting information

Fig S1

Fig S2
