# Supplementary figures and images for: SIRT1 is involved in adrenocortical cancer growth and motility
Source: J Cell Mol Med. 2021 Mar 2;25(8):3856–69. doi: 10.1111/jcmm.16317 (PMC8051751; doi:10.1111/jcmm.16317)

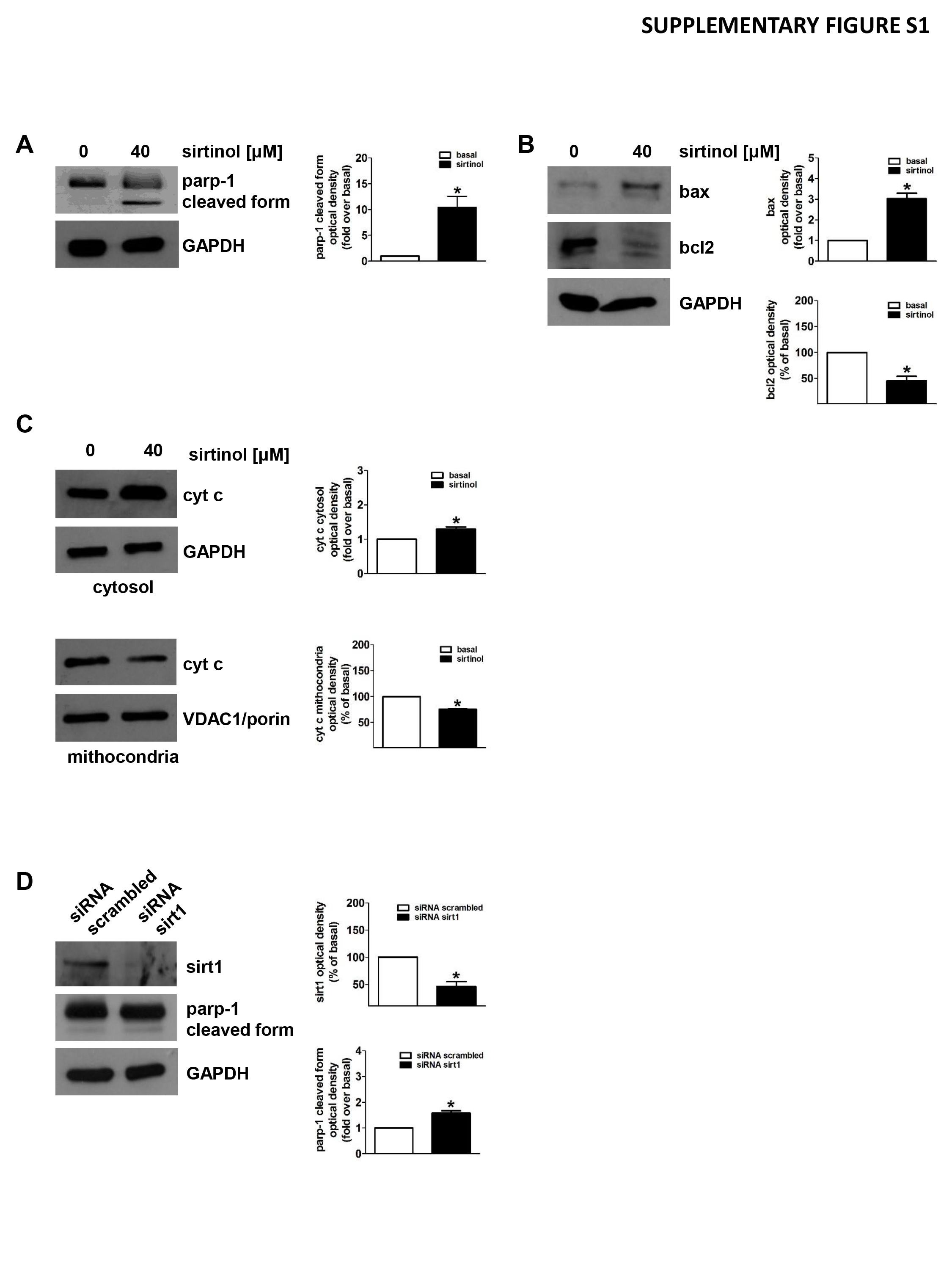

Supplement: Supplementary file 1 — Fig S1 [file JCMM-25-3856-s001.tif]

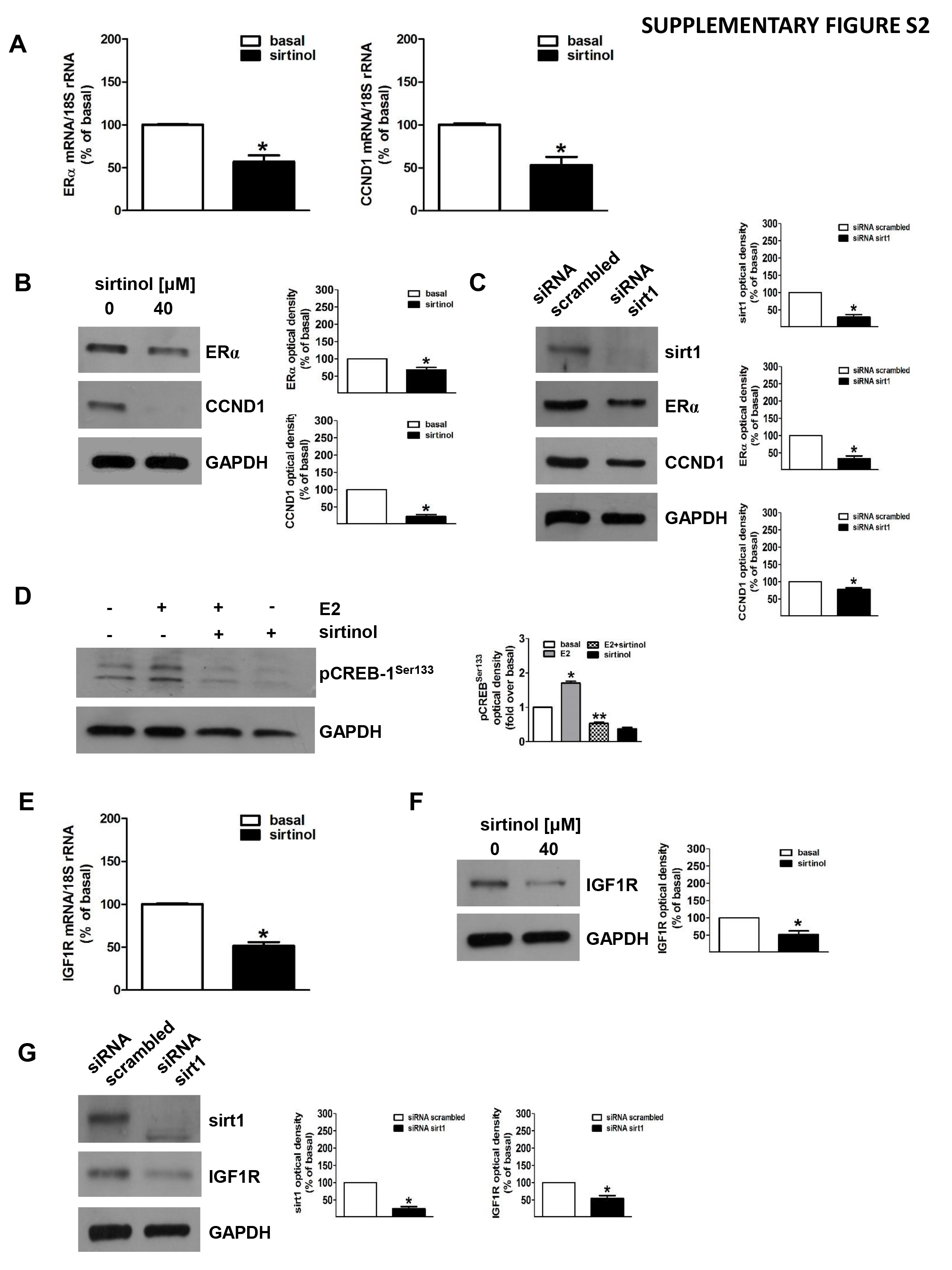

Supplement: Supplementary file 2 — Fig S2 [file JCMM-25-3856-s002.tif]
